# Experimental feasibility of dual-energy X-ray tomography for two-phase density analysis in bentonite during water infiltration

**DOI:** 10.1038/s41598-025-22585-z

**Published:** 2025-11-05

**Authors:** Janne Yliharju, Tero Harjupatana, Enni Rajala, Joni Tanttu, Arttu Miettinen

**Affiliations:** University of Jyväskylä, Department of Physics, Nanoscience Center, Jyväskylä, Finland

**Keywords:** Dual-energy CT, Density measurement, Bentonite, Water, Beam hardening, Scattering, Engineering, Materials science, Techniques and instrumentation

## Abstract

The feasibility of dual-energy X-ray computed tomography (DECT) with experimentally implemented scatter and beam hardening corrections to quantitatively determine the 4D (3D spatial with time) evolution of partial densities of bentonite and water was examined. Compacted bentonite samples were imaged using an X-ray microtomography system with various X-ray spectra before and after two days of water infiltration. The effects of scattering and beam hardening in the projection images were corrected using beam-stop array measurements and signal-to-thickness calibration. A post-reconstruction material decomposition (MD) technique was applied to obtain the partial density distributions of bentonite and water, from which the water content distributions were subsequently derived. The results were validated by physically slicing partially saturated bentonite samples and measuring the water content of the slices gravimetrically. Additionally, a previously developed deformation measurement-based X-ray tomography method was used to derive reference results. Comparing the results from MD with those from the other techniques demonstrated that DECT can yield quantitative estimates of 4D water content distribution in bentonite with reasonable accuracy in this experimental set-up. When testing the impact of correction methods on the results, it was found that both scattering and beam hardening must be corrected, as the post-reconstruction MD is sensitive to errors in spectral measurements.

## Introduction

The quantitative spatio-temporal data on solid and water distributions allows for an in-depth study of moisture-dependent properties of materials and water transport processes. Monitoring the distribution of dry density is crucial, along with water movement, especially when wetting or drying of materials leads to geometric changes, i.e., swelling or shrinking. For instance, wood-based building materials^1,2^ and composite materials^3,4^ exhibit moisture-dependent properties. Additionally, the moisture content of materials is a vital property in geotechnical engineering^5,6^ and plays an essential role in the food industry^7^. To accurately model the behaviour of materials with moisture-dependent properties, experimental data is required to determine the model parameters and validate its accuracy.

Bentonite, a natural clay material consisting mainly of swelling montmorillonite, is a prime example of a material where water transport and the resulting deformations are complex^8^. Bentonite is used in various industries and civil engineering applications^9^. For example, it can be used as a sealant, isolating hazardous materials from the surrounding environment. This type of application is found in geological final disposal for spent nuclear fuel. In various disposal concepts^10^, the radioactive waste is isolated from the biosphere using a multi-barrier system, where a bentonite barrier between the waste canisters and the bedrock is a critical component. It is essential to have a comprehensive understanding of the water transport and hydro-mechanical behaviour of bentonite to design a bentonite barrier system that fulfils the required safety functions.

Water transport in bentonite can be monitored using various techniques that differ in spatial and temporal accuracy. The gravimetric method involves weighing physically sliced subsamples before and after oven drying^11–13^. While this method is straightforward and relatively accurate, it is destructive and requires multiple replicate samples to provide a reasonable number of data points over time. In some studies, multiple sensors monitoring dielectric properties or relative humidity have been used to track changes in water content. However, these indirect measurements necessitate careful calibration and, in some instances, additional information about the properties of bentonite^14–17^. Non-destructive 3D imaging methods such as nuclear magnetic resonance imaging^5,18,19^ (NMRI) and neutron tomography^18,20,21^ (NT) are sensitive to water and are therefore valuable for studying water transport. However, NMRI can be affected by ferromagnetic accessory minerals commonly present in bentonite. Furthermore, the limited availability of NT imaging facilities worldwide presents a practical challenge.

X-ray tomography (CT) is an alternative 3D imaging technique to NMRI and NT. Typically, several hundred or thousands of X-ray images are taken from different angles around the sample. These images are then used to reconstruct a 3D map of the linear attenuation coefficient (LAC), which depends on the elemental composition and mass density of the sample material, as well as the X-ray energy. CT is widely used in various fields to characterise the internal structure of samples^22,23^. Typical analyses performed using CT focus on morphological characteristics, such as pore size distribution. In addition to characterising the properties of a sample at a specific state, dynamic processes can be monitored using 4D CT, which adds time to 3D spatial imaging^24,25^. The process must, however, be slow enough compared to the duration of a single CT scan to avoid motion artefacts. As CT is only moderately sensitive to water compared to NMRI and NT, quantifying the water content distribution in a material is challenging. In the case of bentonite, additional difficulty arises from its swelling behaviour, which needs to be considered, even when the sample is kept under constant total volume conditions. To address these issues, a CT-based method for swelling materials, incorporating deformation measurement as well as careful correction and calibration techniques, was developed in previous studies^26–28^. This method also serves as a reference technique in this study and is referred to as the reference CT method from now on.

Spectral or multi-energy CT is an advancement in the CT field that utilises the material-specific energy dependence of LAC, enabling the identification and quantification of materials^29^. Energy information can be obtained at the X-ray source or detector level, e.g., by adjusting the voltage and filtering of the X-ray tube. A key advancement in laboratory settings is the development of photon-counting detectors (PCDs)^30^, which differentiate photons based on their energy.

Dual-energy CT (DECT) is a common approach within spectral CT, where two tomographic images are captured using different energy spectra. Previously, DECT, using a medical CT scanner, successfully differentiated soil phases: water, air, and solid contents, in a stable state^31^. The weighted means of the volumetric portions of phases and the dry bulk densities corresponded closely with the known composition of the samples and the weighing results. Also, dual-energy transmission measurements using radionuclides^32^ and synchrotron radiation^33^ have been performed to monitor wetting processes. These measurements yielded promising results for 1D information of water content and dry densities during wetting experiments with bentonite powder, a bentonite-sand mixture, and a soil sample. Therefore, DECT has the potential to monitor water transport in bentonite without the need for the challenging deformation measurement required by the reference CT method. However, this assumes that there are no deformations at the spatial scale of imaging resolution between the two CT images captured with different spectra. If the process is fast compared to the imaging time, or the spatial resolution is high enough, it may be necessary to account for displacements in DECT analysis as well. Furthermore, it is important to note that DECT analysis involves solving an inverse problem, making it sensitive to noise and image artefacts, such as those caused by beam hardening and X-ray scattering.

Various methods have been developed to extract material-specific information from DECT. In this study, a process commonly referred to as material decomposition (MD) is employed. Many MD methods rely on breaking down the total LAC of a sample using basis materials^34–36^ (components of the mixture) or by analysing two main interactions^35,37^ in the typical X-ray imaging energy range: the photoelectric effect and Compton scattering. MD can be done pre-reconstruction^37–39^ (in projection space), post-reconstruction^34–36,40^ (in image space), or through a combination of tomographic reconstruction and MD in a single step^41^. One advantage of pre-reconstruction MD is its ability to incorporate the polychromatic energy spectrum of the X-ray source along with the energy-dependent response of the detector. In theory, this approach eliminates measurement errors caused by beam hardening^36,37,39^, a phenomenon where the energy spectrum within the sample varies with position. Such position dependence presents a challenge for post-reconstruction MD, which assumes that the effective energy of the spectrum remains constant throughout the sample. As a result, post-reconstruction MD requires a beam hardening correction. On the other hand, an advantage of post-reconstruction MD is its straightforward implementation. In pre-reconstruction MD and one-step methods, accurately determining the X-ray spectrum and detector response necessitates an advanced theoretical model or experimental characterisation^41^. However, while quantitative post-reconstruction MD does not require that information, calibration of LACs or mass attenuation coefficients is necessary^36^. Additionally, X-ray scattering is seldom incorporated into the MD despite its potential to create artefacts^42–47^. It is essential to consider scattering in measurements, either through theoretical simulations or experimental methods, especially if the objective of the MD is quantitative measurements of the properties of the sample.

This study aims to evaluate the feasibility of DECT post-reconstruction MD to quantitatively determine the partial densities of bentonite and water in 3D, both in a stable initial state and during (relatively slow) water infiltration. In the DECT method of this work, beam hardening is corrected using a two-step signal-to-thickness calibration based on reference plate measurements, and scatter is removed from the images using a beam-stop array. The results given by DECT are compared to those obtained by physically slicing the sample and the reference CT method. The study also assesses the impact of correction methods for beam hardening and X-ray scattering.

## Materials and methods

### Samples and infiltration tests

**Fig. 1:**
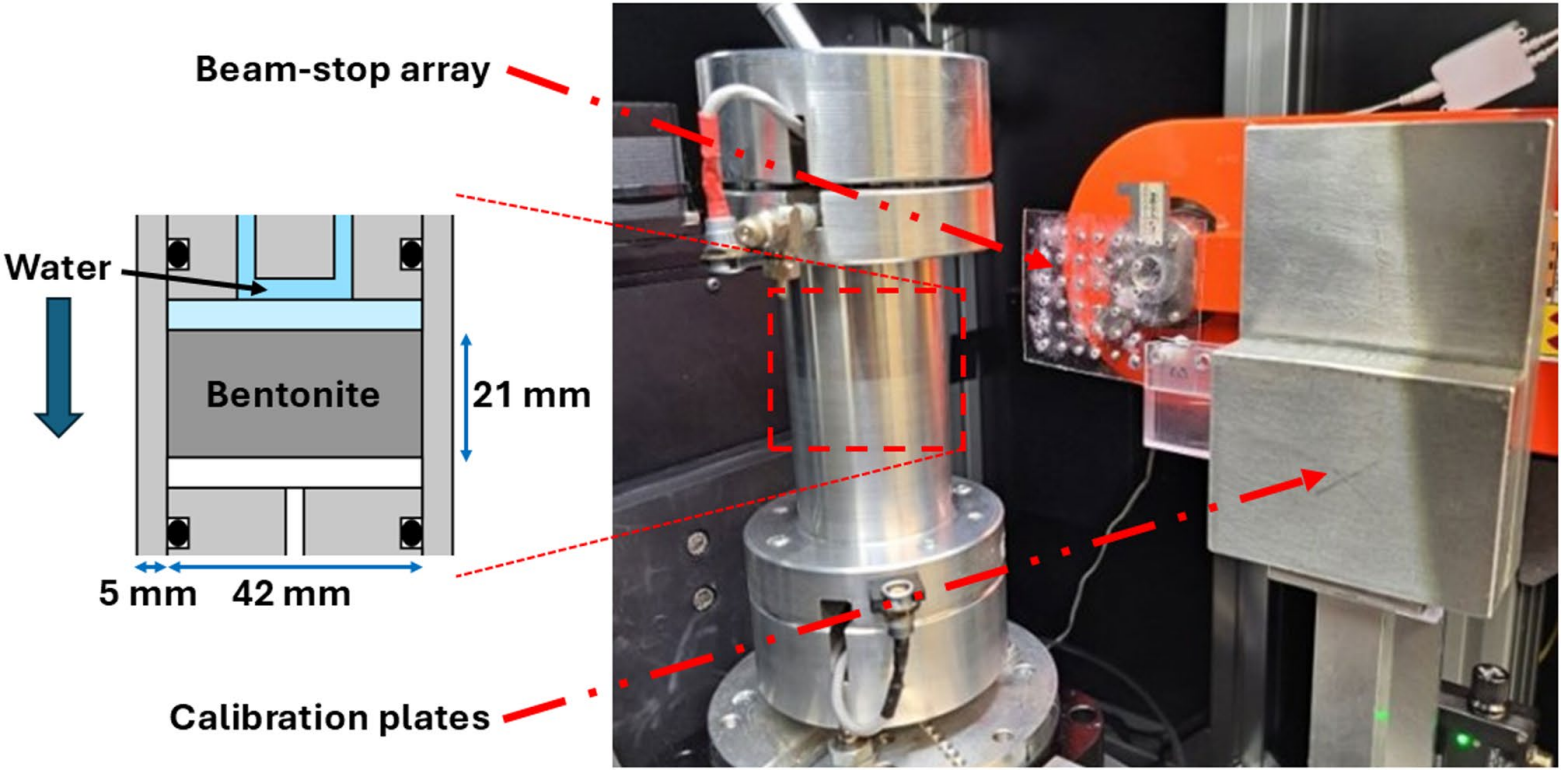
Sample cell for DECT in JTomo microtomograph. The aluminium sample cell and the beam-stop array are positioned in front of the X-ray tube. The two aluminium reference plates used in the dynamic signal-to-thickness calibration are visible on the right side. The schematic cross-sectional enlargement of the sample chamber illustrates the sample and infiltration geometry. The detector is not shown in the photograph.

The clay material used in the tests was Wyoming sodium bentonite (Bara-Kade), provided by Posiva Oy (Eurajoki, Finland) and used without further purification. Thorough characterisation of the material can be found elsewhere^48^. Two similar cylindrical samples, DE1 and DE2, were uniaxially compacted from granular bentonite material. Each sample had a diameter of 42 mm, a height of 21 mm, a dry density of 1.58 g/cm^3^, and an initial water content of 12.0%. The samples were placed inside aluminium sample cells, where they were kept at a constant volume (Fig. 1). In infiltration tests, the samples were wetted from the top end through a porous filter disc (pore diameter 10–16 μm) using distilled water. A similar filter disc at the bottom end allowed for releasing pore air. The tests, for which the complete saturation would take approximately a few weeks, were ended after two days to obtain a non-uniform water content distribution. DECT scans (see below) were performed before the infiltration test in the initial state and after two days of wetting. After the tests, the samples were removed from the sample cell and cut into 2 mm slices in the transverse direction. The water content of the slices was determined gravimetrically by weighing them before and after oven drying at 105 °C for 24 h.

### DECT imaging protocol

CT scans were performed at the University of Jyväskylä (X-ray tomography laboratory) using an in-house built JTomo microtomograph (Fig. 1), which is equipped with a 40–150 kV microfocus X-ray tube (Hamamatsu L12161-07), a flat-panel detector (Teledyne DALSA Shad-o-BOX, 146 mm × 114 mm, 7 MP), and a four-axis sample stage. Each scan consisted of 721 X-ray projection images $\:I$, each of size 1470 × 1152 pixels, taken during continuous 360° rotation. Dark images $\:{I}_{d}$ (signal from the camera without X-rays) and bright images $\:{I}_{0}$ (signal from the camera with X-rays but without a sample) were taken before and after each scan and interpolated over time for the standard flat-field correction, which converts the image signal to transmittance as

1 $$\:T=\frac{I-{I}_{d}}{{I}_{0}-{I}_{d}}.$$

**Fig. 2:**
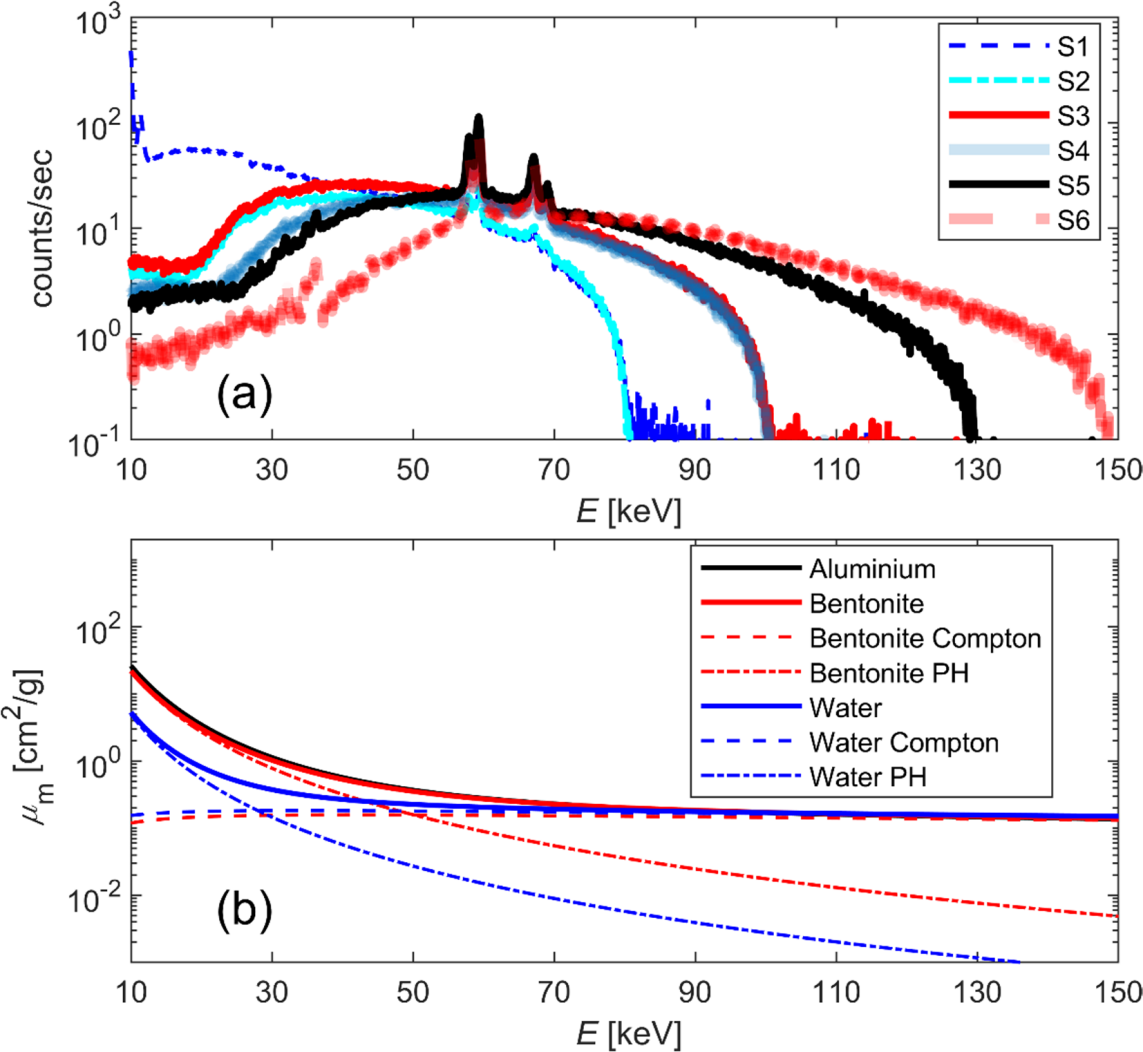
X-ray spectra and mass attenuation coefficients of the materials used. (**a**) The X-ray spectra used for testing the dual-energy CT method (see tube settings in Table 1) were measured with an Amptek XR-100T-CdTe X-Ray and Gamma Ray Detector. (**b**) The mass attenuation coefficients for bentonite, aluminium, and water as a function of energy. For bentonite and water, the contributions of the two most significant interaction mechanisms, Compton scattering (Compton) and the photoelectric effect (PH), are also presented. The plot for bentonite is based on the experimentally determined composition^49^ of montmorillonite (the main mineral in bentonite) in Wyoming sodium bentonite, which is similar to the material studied here. The mass attenuation coefficient values were obtained from the NIST XCOM database^50^.

A source-to-sample distance of 209 mm and a source-to-camera distance of 434 mm resulted in a pixel size of 48 μm. To minimise cone beam artefacts, two overlapping scans centred at the top and bottom ends of the sample were acquired and subsequently stitched together after reconstruction. Since the exact positioning of the top and bottom ends of the samples within the sample cell may vary, the imaging coordinates were updated for each sample separately. To test different spectral pairs for DECT analysis, scans were repeated using various X-ray spectra by changing the tube voltage and filtering (Table 1; Fig. 2a). The tube current and exposure time were adjusted to obtain approximately the same signal level at the camera for all spectra.

**Table 1:** Specifications for different X-ray spectra.

Spectrum	Sample	X-ray tube			Exposure [ms]	$\:{T}_{\text{min}}\:$[%]
		Filter	Voltage [kV]	Current [µA]		
S1	DE2	No	80	220	400	1.9
S2	DE2	2.1 mm glass	80	400	600	4.3
S3	DE1	2.1 mm glass	100	300	450	6.5
S4	DE1	3.3 mm glass	100	400	500	7.8
S5	DE2	5.0 mm glass	130	260	500	11.2
S6	DE1, DE2	1.0 mm Cu	150	350	500	16.9

The table lists the spectra used in this study, along with parameters affecting the energy interval (filtering and voltage) and signal level (current and exposure time per projection image). The last column shows the measured minimum transmittance through the bentonite sample cell.

In this study, signal-to-thickness calibration^51,52^ (STC) was used to correct beam hardening, but unlike the conventional method, it was applied in two steps. The static STC first corrects the general trend of beam hardening, while the subsequent dynamic STC addresses the spatio-temporal variations in the X-ray spectrum and the response of the detector. For the static STC, nine aluminium plates with thicknesses in the range of 2.0–49.3 mm were imaged separately from the rest of the protocol, using the same energy spectrum and camera settings as those used in the corresponding tomographic scans. The calibration employed a fifth-order polynomial

2 $$\:{x}_{s}={\sum\:}_{k=1}^{5}{a}_{k}{\text{log}}^{k}\left(T\right),$$

which converts the measured transmittance *T* (Eq. 1) into the equivalent thickness of aluminium $\:{x}_{s}$ pixel-wise. The coefficients $\:{a}_{k}$ were determined by fitting Eq. (2) to the average transmittances of the reference plates and their corresponding thicknesses. In the dynamic STC, the equivalent thicknesses $\:{x}_{s}$ obtained from the static STC were further corrected pixel-wise using a quadratic polynomial

3 $$\:{x}_{\text{d}\text{y}\text{n}}={A}_{1}{x}_{s}+{A}_{2}{x}_{s}^{2}.$$

The coefficient matrices $\:{A}_{1}$ and $\:{A}_{2}$ were calculated pixel-wise using X-ray images of two aluminium reference plates (19.6 mm and 39.2 mm thick) attached next to the rotation stage (Fig. 1). These images, taken before and after each scan, were converted to equivalent thickness values through Eqs. (1) and (2). Aluminium was chosen as the reference material because the energy dependence of its mass attenuation coefficient is similar to that of bentonite and matches that of the aluminium sample cell (Fig. 2b).

For scatter correction (SC), a beam-stop array (BSA) was attached to an additional three-axis stage (Fig. 1). The BSA was constructed by glueing 6 × 6 lead spheres (4 mm in diameter) onto an acrylic plate (69 mm wide, 92 mm high, and 1 mm thick), arranged in a regular grid with a centre-to-centre distance of 10 mm. To measure the contribution of scattering, the BSA was placed between the source and the sample at 77 mm from the source. The total signal $\:I$ recorded at the camera was assumed to be the sum of the primary signal $\:{I}_{p}$ and the secondary signal $\:{I}_{s}$ (i.e., scattering and charge spreading in the detector). It was also assumed that the BSA provides measurements of secondary signals at the locations of the spheres, as the lead spheres block the primary signal. Instead of using interpolation, the BSA was moved in 1 mm increments to cover the entire image area, acquiring an image after each move. All images taken with the BSA (110 in total) were then combined to create an estimate of the scattering field $\:{I}_{s}$. The measurement of the secondary signal was performed for the bright and reference plate images, as well as for one projection image of each scan. When applying the SC, the corresponding $\:{I}_{s}$ estimate was subtracted from the image to be corrected. Next, flat-field correction (Eq. 1) was carried out on the projections and reference plate images using the SC-corrected images. For the projection images, the scattering field from the first rotation angle was applied to all projection images in the scan. This approach was feasible due to the rotational symmetry of the sample and infiltration geometries, resulting in significant time savings in the imaging protocol.

The imaging protocol, including two aluminium plate images for dynamic STC, bright images, tomographic scans, and corresponding SC measurements, was executed automatically using Python scripting, eliminating the need to turn off the X-ray source between steps, thus avoiding errors in signal levels caused by the need to wait for the equipment to stabilise after each restart. The exact duration of the protocol depended on the exposure time of the projections, but in all cases listed in Table 1, it was approximately one hour. Acquiring the projections (2 × 721), including estimating the secondary signal with the BSA, took approximately 20 min. The corrected projections (converted into $\:{x}_{\text{d}\text{y}\text{n}}$ after the SC and the two-step STC) were used to reconstruct the 3D CT images using the Feldkamp-Davis-Kress formulation of the filtered back-projection algorithm implemented in the pi2 software^53^. All CT images were binned down to an isotropic voxel size of 382 μm to increase the signal-to-noise ratio for the DECT analysis. No pre-processing of CT images, such as smoothing filters, was performed before the DECT material decomposition.

### Post-reconstruction material decomposition

Density measurements rely on the linear combination

4 $$\:\mu\:\left(E,\overrightarrow{r}\right)={c}_{b}\left(E\right){\rho\:}_{b}\left(\overrightarrow{r}\right)+{c}_{w}\left(E\right){\rho\:}_{w}\left(\overrightarrow{r}\right),$$

where the linear attenuation coefficient $\:\mu\:$, obtained from CT images and dependent on energy *E* and position $\:\overrightarrow{r}$, separates into the energy-dependent mass attenuation coefficients $\:{c}_{b}$ and $\:{c}_{w}$, and the position-dependent partial densities $\:{\rho\:}_{b}$ and $\:{\rho\:}_{w}$ in a mixture of bentonite (b) and water (w). In reality, the mass attenuation coefficients $\:{c}_{b}$ and $\:{c}_{w}$ depend on the location. However, in this study, both beam hardening and scatter corrections are used to support the assumption in Eq. (4) regarding the uniform applicability of the mass attenuation coefficients without position dependence. The partial density of a mixture component is defined as the mass of that component within a control volume, such as a voxel (volumetric pixel), divided by the volume of that control volume. The partial density of bentonite is also known as dry density.

In post-reconstruction MD based on DECT, there are two equations

5 $$\:\left\{\begin{array}{c}\mu\:\left({E}_{1},\overrightarrow{r}\right)={c}_{b}\left({E}_{1}\right){\rho\:}_{b}\left(\overrightarrow{r}\right)+{c}_{w}\left({E}_{1}\right){\rho\:}_{w}\left(\overrightarrow{r}\right)\:\\\:\mu\:\left({E}_{2},\overrightarrow{r}\right)={c}_{b}\left({E}_{2}\right){\rho\:}_{b}\left(\overrightarrow{r}\right)+{c}_{w}\left({E}_{2}\right){\rho\:}_{w}\left(\overrightarrow{r}\right),\end{array}\right.$$

one for each effective energy $\:{E}_{i}$. This group of equations is examined for each voxel and can be written in a matrix form $\:\overrightarrow{\mu\:}=C\overrightarrow{\rho\:}$. A unique solution for the density vector $\:\overrightarrow{\rho\:}=[{\rho\:}_{b},\:{\rho\:}_{w}]$ can be found if the determinant of the mass attenuation coefficient matrix $\:C$ is non-zero, which holds if

6 $$\:\frac{{c}_{b}\left({E}_{1}\right)}{{c}_{b}\left({E}_{2}\right)}\ne\:\frac{{c}_{w}\left({E}_{1}\right)}{{c}_{w}\left({E}_{2}\right)}.$$

Equation (6) indicates that to accurately determine densities, the ratios of the mass attenuation coefficients of the basis materials must differ between the two energy spectra examined in DECT. If they do not, the matrix $\:C\:$becomes ill-conditioned and leads to unreliable solutions. As shown in Fig. 2b, the energy dependence of the mass attenuation coefficients of bentonite and water differ within the energy range used in this study. Based on this theoretical behaviour, DECT is applicable to the bentonite-water mixture. However, the accuracy of the post-reconstruction DECT MD relies on the precision of the experimental measurements of the linear and mass attenuation coefficients.

Since DECT density analysis requires known mass attenuation coefficients of the mixture components, an experimental calibration was applied instead of relying on theoretical values. This calibration utilised the average partial densities of bentonite and water, along with the LACs, over the entire sample in both the initial and final states of the test. In the calibration of mass attenuation coefficients at the effective energy $\:{E}_{i}$, two equations were used

7 $$\:\left\{\begin{array}{c}{\widehat{\mu\:}}_{0}\left({E}_{i}\right)={c}_{b}\left({E}_{i}\right){\widehat{\rho\:}}_{b0}+{c}_{w}\left({E}_{i}\right){\widehat{\rho\:}}_{w0}\:\\\:{\widehat{\mu\:}}_{1}\left({E}_{i}\right)={c}_{b}\left({E}_{i}\right){\widehat{\rho\:}}_{b1}+{c}_{w}\left({E}_{i}\right){\widehat{\rho\:}}_{w1},\end{array}\right.$$

corresponding to the initial (0) and final (1) states of the experiment. In Eq. (7), the spatial averages are indicated by the notations $\:\widehat{\mu\:},\:{\widehat{\rho\:}}_{b},$ and $\:{\widehat{\rho\:}}_{w}$. The initial densities were determined based on the water content of the bentonite powder and the total mass of the sample. The final densities were measured after removing the sample from the sample cell using its total mass, average water content, and the dimensions of the sample determined from CT images.

The Moore-Penrose pseudoinverse matrix $\:{C}^{+}$ (calculated using MATLAB’s *pinv* function) was used to solve the densities from Eq. (5) for each voxel as $\:\overrightarrow{\rho\:}={C}^{+}\overrightarrow{\mu\:}$. The mass attenuation coefficients were solved from Eq. (7), where average LACs and densities are known, using the same approach.

### Classification of image elements based on dual-energy ratio

By studying the material-specific energy dependency of the LAC, voxels can be classified into different material categories. Regarding the characterisation of bentonite and its properties, this is potentially of importance since this natural clay material contains accessory minerals such as pyrite, gypsum, and quartz, whose elemental compositions and hydro-mechanical properties differ from those of the surrounding montmorillonite-rich clay matrix. Although these accessory minerals also contain the same elements as montmorillonite (e.g., Si and Ca), their relative fractions differ from those of montmorillonite. Analysing the dual-energy ratio

8 $$\:k=\frac{\mu\:\left({E}_{1}\right)}{\mu\:\left({E}_{2}\right)}=\frac{\mu\:\left({E}_{1}\right)/\rho\:}{\mu\:\left({E}_{2}\right)/\rho\:}=\frac{c\left({E}_{1}\right)}{c\left({E}_{2}\right)}$$

of voxels allows for the examination of variations in the elemental composition of bentonite.

The dual-energy ratio $\:k$ was calculated for each voxel in the 3D image. The average and the standard deviation of *k* in the bentonite-water region were determined, and voxel values that differed by more than two standard deviations from the average were classified as materials other than the bentonite-water mixture. The latter class represents aluminium walls, air surrounding the sample cell, other materials present in the set-up, accessory minerals (if their composition significantly differs from the average composition of bentonite), or image artefacts. Only the voxels classified as the bentonite-water mixture were included in the MD density analysis.

### Reference CT method

The results from the DECT method were compared to those from the reference CT method, which is not covered in detail here, as comprehensive descriptions are available in the literature^26–28^. The method involves comparing CT images of the reference state, where the water distribution is known, to other states. The difference image can be calibrated to reflect the partial density distribution of water. For swelling materials such as bentonite, aligning the material points between images using the deformations measured through digital volume correlation is essential to ensure proper comparison. Additionally, during the wetting process, the density distribution of the solid component is updated based on the divergence of the displacement field, which describes the relative volume change. Therefore, accurate deformation measurement plays a crucial role in the reference CT method. This method provides the final results as partial density distributions of solid and water. Here, CT images taken with spectrum S6 and applying both the SC and two-step STC were used for the reference CT method, with a voxel size of 191 μm to ensure accurate deformation measurement. For analysing density distributions, CT images were binned to the same voxel size as in the DECT analysis, 382 μm, to facilitate comparison between the methods.

## Results

### Partial densities of bentonite and water based on DECT material decomposition

**Fig. 3:**
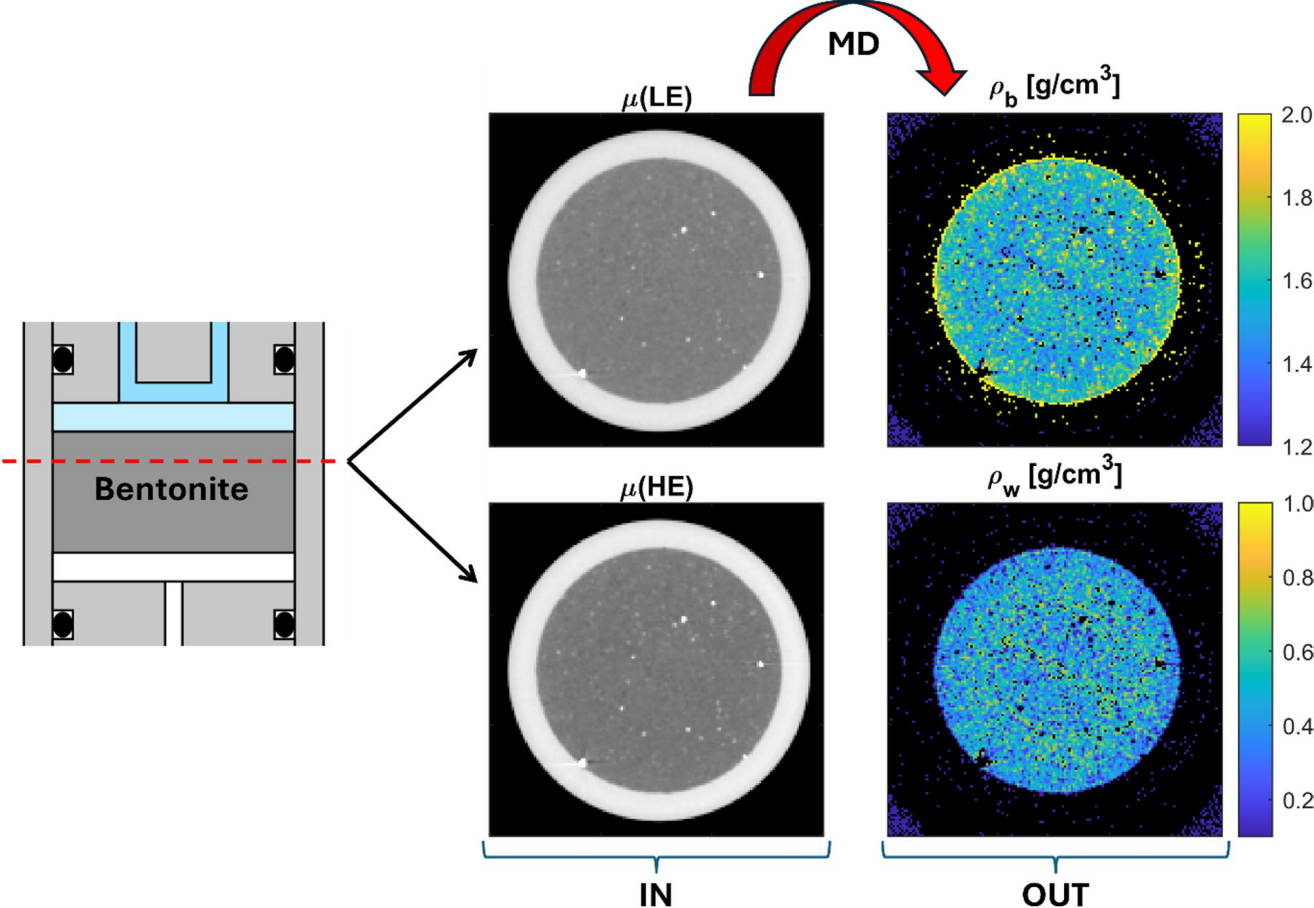
Illustrative examples of results from material decomposition (MD). The CT images obtained using low-energy (LE) and high-energy (HE) spectra, S3 and S6, respectively, serve as the input (IN) for the MD. The output (OUT) of MD consists of density maps for the partial densities of bentonite and water. This illustration shows individual tomographic cross-sections, with a red dashed line in the schematic of the sample cell indicating the approximate position of these cross-sections. In the density maps, black represents voxels that correspond to materials other than bentonite or water.

Figure 3 shows examples of the results for sample DE1 in the final state. Two CT images acquired with low energy (LE, S3) and high energy (HE, S6) spectra were used as the input of the analysis, yielding 3D density maps of bentonite and water. The density maps were created by examining the entire 3D volume voxel by voxel. Each voxel was first classified, and then Eq. (5). was applied to each voxel identified as part of the bentonite-water mixture. In the density maps shown in Fig. 3, black represents voxels identified as materials other than the bentonite-water mixture. The surrounding air and the higher-density accessory minerals, indicated by white spots in the clay in the X-ray CT images, are mainly categorised correctly based on visual comparison of the images and density maps. Also, the streak artefacts originating, for example, from a high-density particle located in the bottom left of the cross-section of the sample are excluded from the density analysis. Some voxels in the aluminium wall of the sample cell are classified as a bentonite-water mixture with high bentonite density and low water density. Notably, the voxels at the interface of bentonite and aluminium wall, partially in bentonite and partially in aluminium, form a yellow ring of high-density bentonite. This classification is reasonable in the presence of measurement errors, as the energy dependence of aluminium closely resembles that of bentonite (see Fig. 2b). Moreover, the partial density values of bentonite and water are reasonable, when comparing to the average values of 1.58 g/cm^3^ and 0.33 g/cm^3^, respectively. Although the original X-ray CT images showed only weak ring artefacts, these artefacts became more apparent in the density maps. This is likely due to the amplification of errors during the inversion of Eq.(5).

**Fig. 4:**
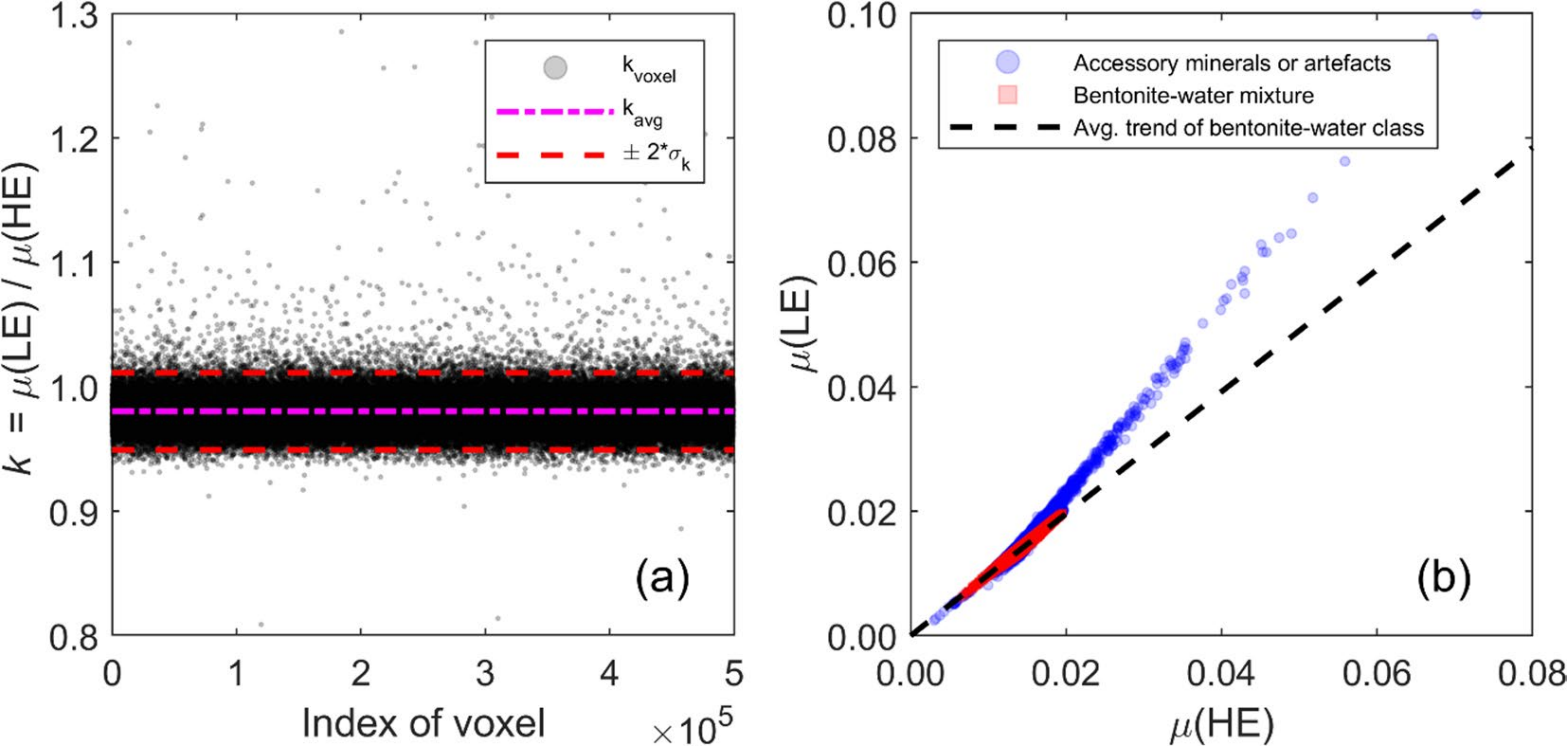
Classification of voxels based on dual-energy ratio. (**a**) The dual-energy ratios of all voxels from sample DE1, using low- (LE) and high-energy (HE) spectra S3 and S6, are shown, along with the limits defined by two standard deviations from the mean. Voxels within the limits are interpreted as a bentonite-water mixture, while those outside this range are classified as accessory minerals or image artefacts. (**b**) The classification results are visualised in a dual-energy plot. In this plot, materials with similar elemental compositions appear along specific lines, with their density determining their position on those lines. Accessory minerals represent a distinct material category that has a different slope (or slopes) compared to the bentonite-water mixture.

The classification step of the MD is illustrated in Fig. 4a, where the dual-energy ratios of all voxels within the bentonite sample are plotted along with the average ratio and limits defined by two standard deviations. The selection criterion was based on visual inspection of the dual-energy ratio distribution. Figure 4b shows a dual-energy plot, where the linear attenuation coefficient from the low-energy CT image is plotted against that from the high-energy image. The average dual-energy ratio of the bentonite-water class is close to unity ($\:k=0.98$), as expected after the STC for materials with a composition similar to aluminium. However, many voxels have significantly larger ratios, likely corresponding to accessory minerals with heavier elements than montmorillonite. In the dual-energy plot, the dual-energy ratio reflects the slope of the line representing a material class with a specific elemental composition. Based on the varying elemental composition of accessory minerals in bentonite and the distribution of dual-energy ratios, the “accessory minerals line” actually comprises multiple lines with different slopes. The total mass density determines the position of a voxel on the line in the dual-energy plot.

The classification step proved important, as without it, areas corresponding to accessory minerals showed unrealistically high dry density values (up to over 40 g/cm³) and negative density values for water. These instances lead to negative water content values (with large magnitudes) and could potentially skew the local averages needed for the method comparisons in the next section. The volume fractions of rejected voxels were approximately 3% for DE1 and 4% for DE2.

### Comparison of DECT material decomposition to reference methods

**Fig. 5:**
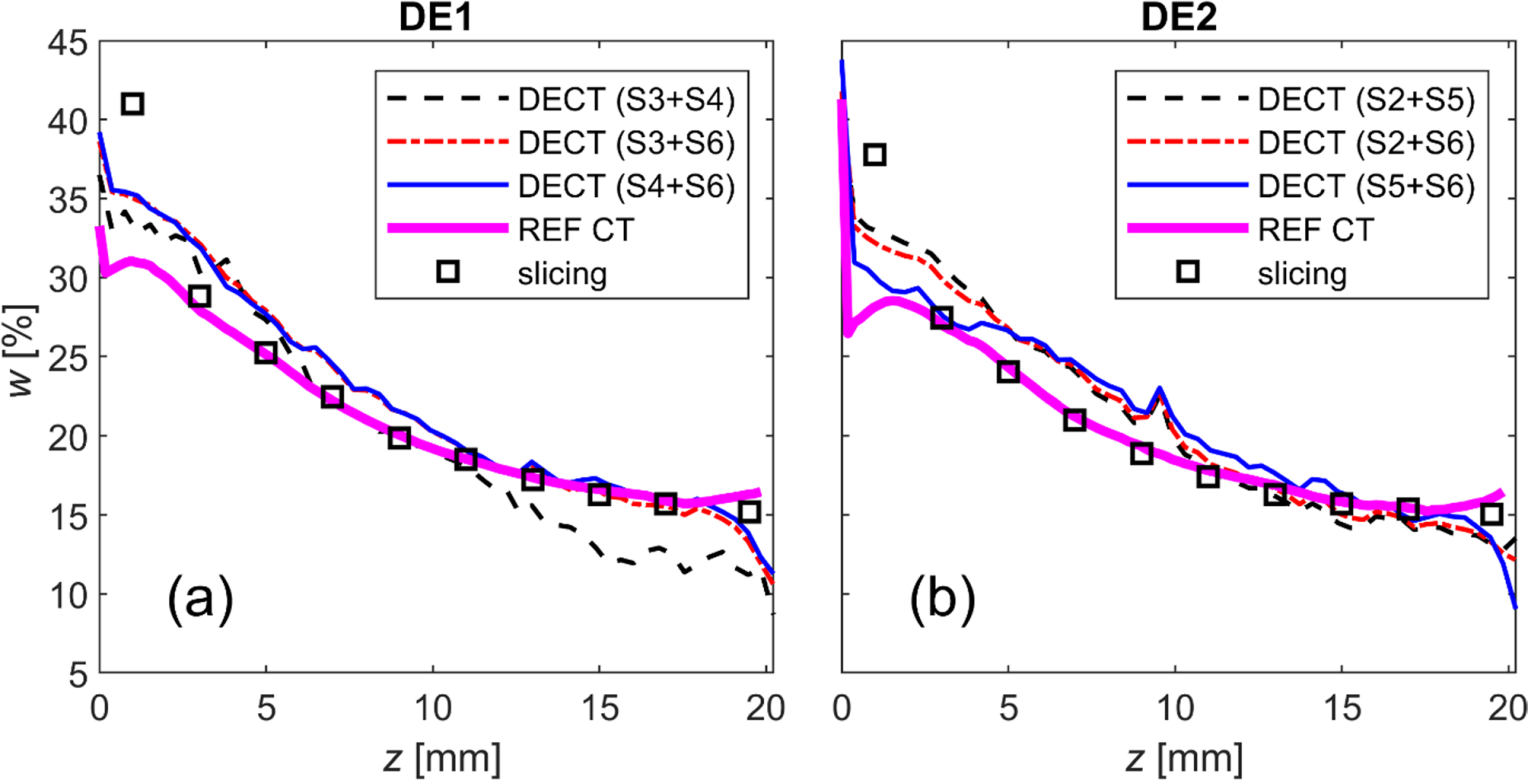
Water content profiles along bentonite samples measured by different methods. Bentonite samples DE1 (**a**) and DE2 (**b**) were identical. The results of dual-energy CT (DECT) are shown with three different spectra pairs, while the reference CT method (REF CT) used a single spectrum (S6). The slicing refers to the gravimetric method conducted after the tests. The details of the energy spectra are provided in Table 1.

To facilitate comparison, the three-dimensional water content distributions, calculated using the MD and reference CT methods, were averaged into one-dimensional axial water content profiles. Figure 5 presents the water content profiles obtained by DECT for three pairs of spectra, alongside results from the reference CT method and gravimetric slicing. Spectrum S1 results are excluded due to very low transmittance through the sample (Table 1), causing artefacts in reconstructions and unreliable density measurements. Overall, the results in Fig. 5 from the different methods are generally consistent. However, compared to physical slicing, the reference CT method is more accurate than DECT, except near the water inlet (at 0 mm). The DECT results using different spectrum combinations show relatively good consistency, but spectra S3 and S4 in DE1 overlap significantly (see Fig. 2a), leading to inaccurate results.

**Fig. 6:**
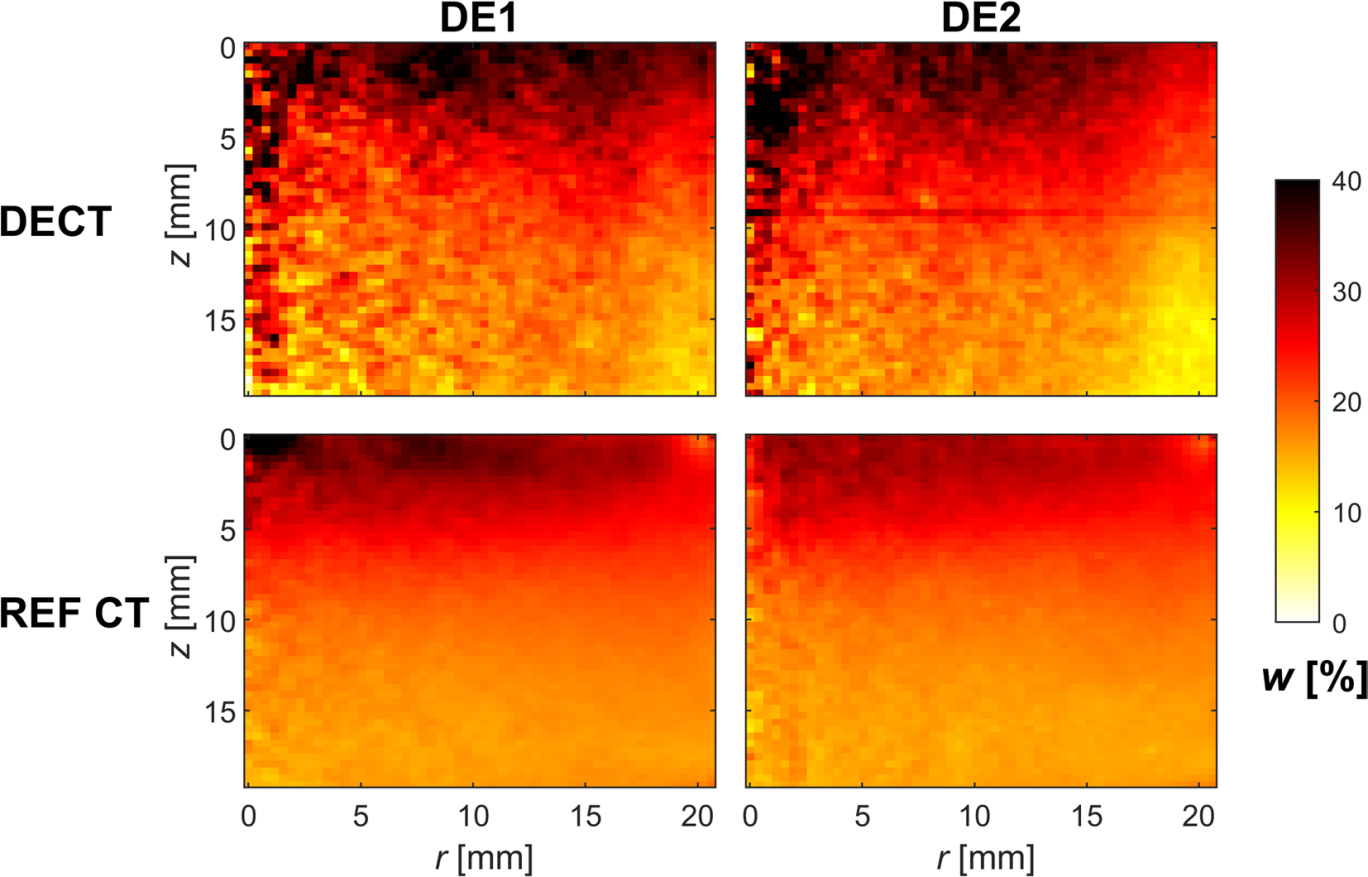
Azimuthally averaged water content distributions in bentonite samples measured by DECT and the reference CT method. The top row displays the results of the dual-energy CT (DECT) method using spectra S4 and S6 for sample DE1 (top left) and spectra S2 and S6 for sample DE2 (top right). The bottom row shows the corresponding results of the reference CT method (REF CT) using spectrum S6.

To better illustrate the water content distributions at different positions within the sample, Fig. 6 presents azimuthally averaged water content distributions in the final state obtained by the DECT and reference CT methods. The results from both methods are generally similar, though the DECT results seem noisier. In this respect, it is important to note that the reference CT method requires deformation measurement (which is not always successful or even possible). The deformations are represented on a lower-resolution grid compared to the image resolution, effectively lowering the spatial resolution and decreasing noise in the result compared to the DECT that is calculated independently for each voxel. Given this, the DECT results are very promising. Additionally, for modelling purposes, high spatial resolution is not always necessary, allowing for filtering or binning of results to reduce noise. Furthermore, the signal-to-noise ratio in azimuthal averages varies with radius, as the number of averaged voxels depends on the radius. However, this effect is consistent for both the DECT and reference CT visualisations, enabling the comparison of the methods. Despite corrections for beam hardening and scattering, a systematic error is observed in DECT results in the lower half of both samples. The DECT results indicate drier areas that are not observed in the results from the reference CT method, particularly in the bottom right corner of the plots. The reason for this effect remains unclear, but it could be related to a higher-degree scattering artefact not corrected perfectly.

### Significance of scatter and beam hardening corrections

**Fig. 7:**
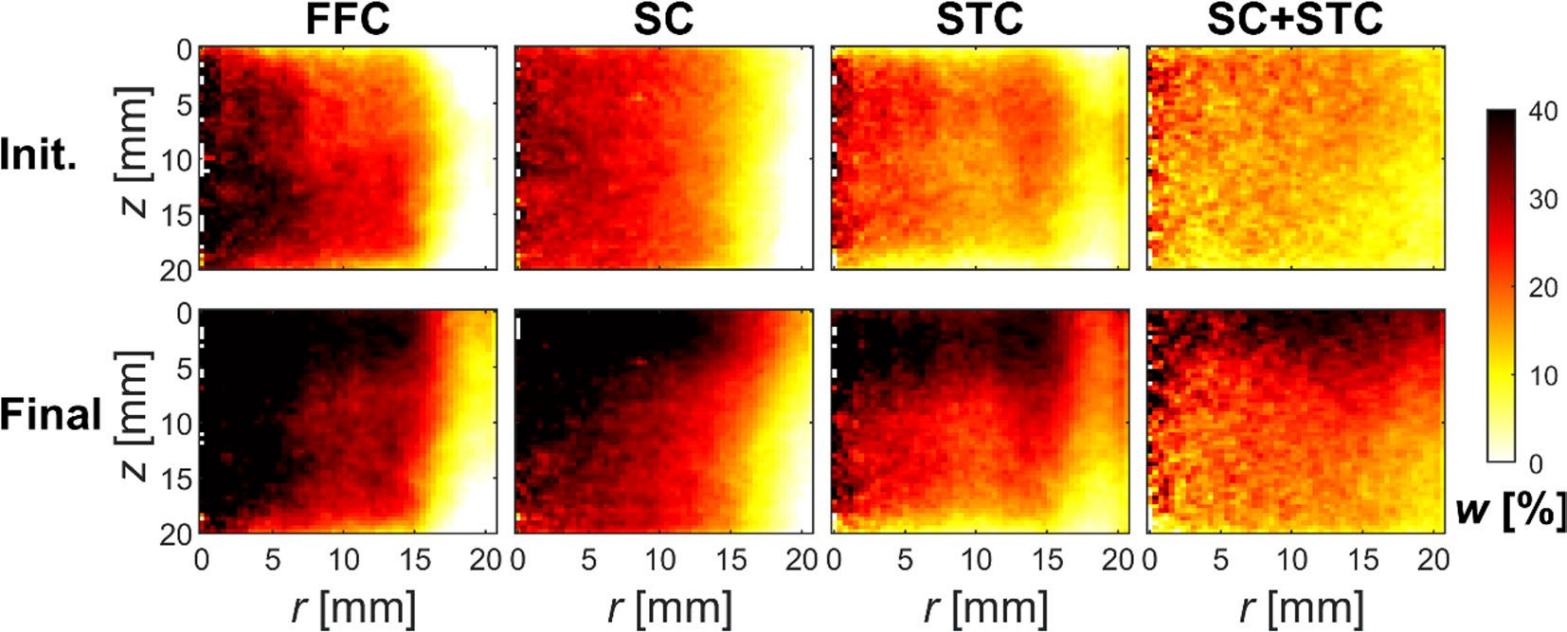
Influence of scatter and beam hardening corrections on DECT results. The panels show azimuthally averaged water content distributions for sample DE1 using spectra S4 and S6 in the initial (top row) and final states (bottom row) with different combinations of correction methods. In the initial state, the water content (*w*) is assumed to be uniform. In the final state, the assessment of corrections is based on a comparison of the results from different approaches with those of the reference CT method (bottom left panel in Fig. 6). The acronym FFC refers to images that have undergone flat-field correction only, while SC denotes scatter-corrected data. STC indicates data corrected only for beam hardening, and the last column represents results combining both scatter correction and beam hardening correction. The results correspond to those in the left column of Fig. 6.

To assess the significance of the scatter and beam hardening corrections in the proposed DECT method, Fig. 7 shows azimuthally averaged water content distributions in the bentonite sample DE1 utilising different combinations of correction methods. The initial water content is assumed to be uniformly at 12%, and SC + STC most closely aligns with this assumed distribution. Additionally, in the final state, SC + STC shows the best agreement with the reference CT method (see Fig. 6), although an increase in water content is visible in the top half of the sample for every correction approach. Using only SC or STC, the water content distributions vary with the radius, particularly evident in the initial state, and generally appear qualitatively incorrect. Furthermore, using only STC results in a decrease in water content near the top and bottom surfaces. Therefore, both beam hardening and scattering present challenges that need to be addressed in the proposed DECT method. The FFC-only approach yields strongly misleading results due to artefacts in the X-ray CT images.

**Fig. 8:**
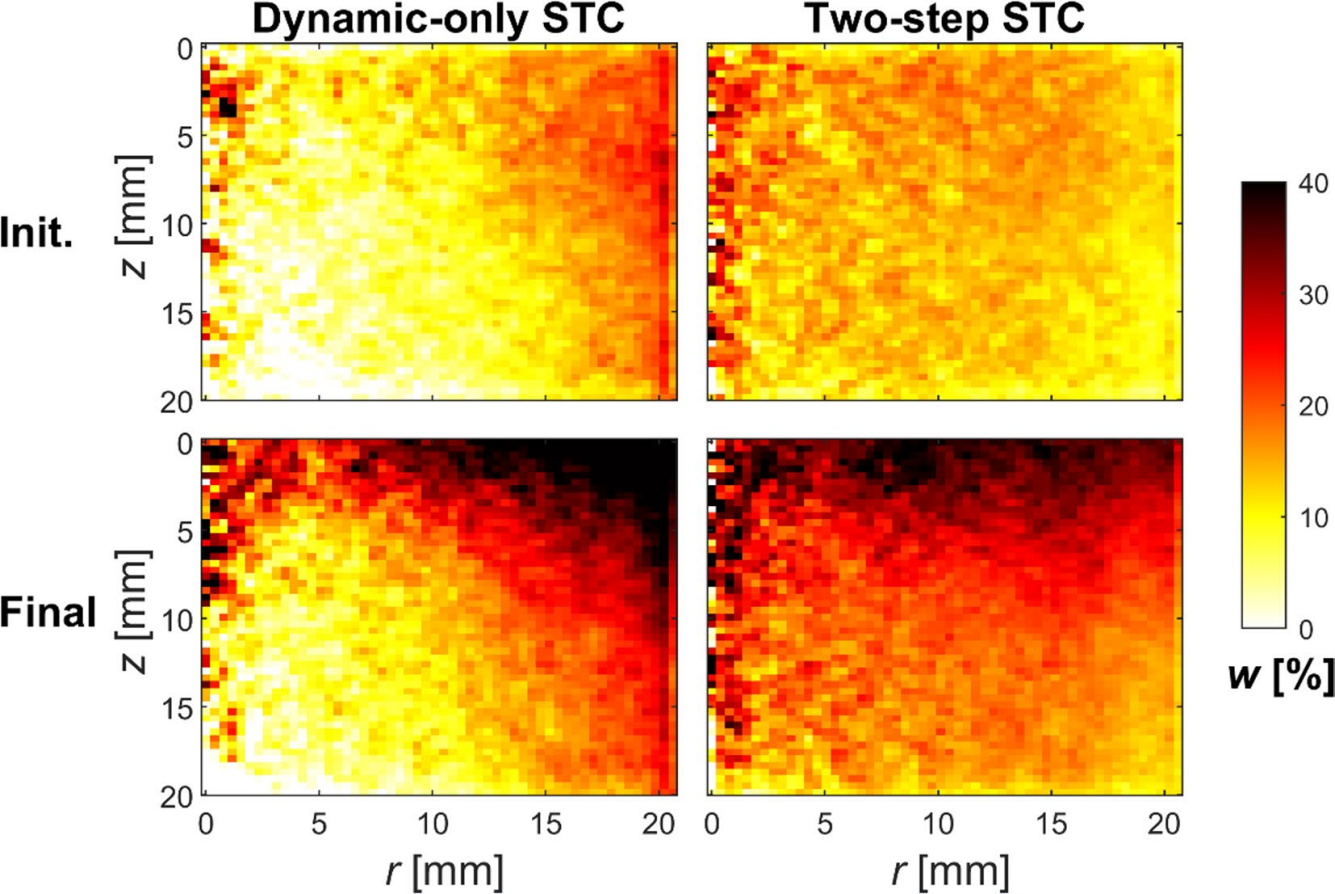
Importance of the static STC in post-reconstruction MD. The panels show azimuthally averaged water content distributions in the initial (top row) and final states (bottom row) for sample DE1 using spectra S4 and S6 and scatter corrected (SC) data. The panels in the left column show results using only the dynamic STC, while the right column panels show results from the two-step STC that combines dynamic and static STC. The water content distribution is assumed to be uniform (*w* = 12%) in the initial state. In the final state, the assessment of the beam hardening correction is based on a comparison of the results from different approaches with those of the reference CT method (bottom left panel in Fig. 6).

The significance of the static STC is illustrated in Fig. 8, which shows the azimuthally averaged water content distributions in both the initial and final states, using only the dynamic STC or the two-step STC. By comparing these results with the assumed uniform distribution in the initial state and with the results from the reference CT method in the final state (see Fig. 6), it became clear that a static STC is necessary. This highlights the need for careful correction of beam hardening and that a quadratic polynomial is insufficient for the materials and X-ray spectra used in this study.

Additionally, the coefficient images$\:\:{A}_{1}$ and $\:{A}_{2}$ for the dynamic STC (Eq. 3) were assessed qualitatively. These matrices were determined for each scan separately, and variations were observed between measurements. As a general trend, it appears that both matrices were more uniform when the scatter correction was applied, and pixel values in $\:{A}_{1}$ were close to one and in $\:{A}_{0}$ they were close to zero. When scatter correction was not applied, the influence of dynamic STC was more pronounced. Detailed quantification of the interplay between dynamic STC and scatter correction is left for future work.

## Discussion

This study examined the applicability of DECT post-reconstruction MD for measuring the partial density distributions of bentonite and water during water infiltration. To compare DECT and reference methods, imaging was conducted both in the stationary initial state to obtain a uniform water content distribution and during water infiltration to achieve a non-uniform water content distribution. Overall, DECT results showed good agreement with those given by the reference methods, indicating its suitability for analysing the bentonite-water mixture and monitoring the water transport process under these experimental conditions. Additional experiments using varying conditions are necessary to enhance the understanding of the applicability of DECT, as this study focused on testing different spectra and thoroughly examining the effects of correction methods under specific conditions. Despite promising results, DECT exhibited significantly higher noise levels compared to the reference CT method. Additionally, DECT provided slightly higher water content values than both the reference CT method and gravimetric slicing. The exact cause of this discrepancy is unknown but may be related to the classification step, where unrealistic density values were rejected. Although the volume fraction of discarded voxels was small, the rejection criteria may have slightly biased the results.

Beam hardening presents challenges in post-reconstruction MD. The results indicate that addressing this phenomenon is crucial for obtaining reliable measurements. In this study, the STC was conducted using a non-standard two-step approach. The dynamic-only correction, which utilised a quadratic polynomial pixel-wise, was insufficient and led to distorted water content distributions. Adding a static STC with a globally applied fifth-order polynomial is critical in the presented methodology. This suggests that spatio-temporal STC for DECT analysis requires calibration measurements with several reference plate thicknesses and characterisation of beam hardening effect using a sufficiently high-degree polynomial. Although the correction could be done in a single step, a two-step correction is easier to implement in practice. Additionally, observations suggest that dynamic STC becomes less critical when both scatter correction and static STC are applied. The advantages of removing dynamic STC and, for instance, substituting it with a more extended warm-up period after changing X-ray spectra, are evident. This approach could simplify implementation and reduce potential noise caused by pixel-wise corrections. However, a longer warm-up period results in extended imaging times, which is not ideal for monitoring dynamic processes using DECT. Dynamic STC is intended to minimise uncertainties related to fluctuations in experimental conditions. However, these fluctuations are likely dependent on the specific device and settings used, and their significance requires thorough characterisation for each particular application.

The significance of scatter correction of projection images for accurate post-reconstruction DECT MD became clearly evident in this study. Similar conclusions have been reached, e.g., in the medical field when quantifying iodine concentration using spectral CT^45^. A beam-stop array used here is a simple method for measuring the scatter field. However, to increase spatial resolution, the array needs to be repositioned step-wise multiple times, requiring numerous images for correction, which extends imaging time. In this study, the sample and the wetting geometry were rotationally symmetric, allowing the scatter field to be measured at a single rotational angle, assuming it is the same for all other angles. This assumption is supported by prior studies indicating that the scattering signal varies smoothly compared to the primary signal^46,47,54^. However, for samples of more complex shapes, the scatter field must be measured at multiple angles, significantly increasing imaging time and potentially limiting the applicability of the method in certain situations.

Each scan was repeated with several spectra to test the significance of different spectral pairs on DECT results. The low transmittance of the lowest energy spectrum S1 caused artefacts and highly unreliable results. Otherwise, the results improved with more distinct spectra, as expected, based on the energy dependencies of the mass attenuation coefficients for bentonite and water (Fig. 2b). In practice, testing every possible spectral pair is not feasible due to the unlimited number of options available. There is no evidence suggesting that any of the pairs utilised in this study represents the best option in terms of the accuracy of the MD. Therefore, further research is required.

Although the reference CT method yielded more accurate results, it requires deformation measurement, which is often challenging and sometimes impossible, such as with homogeneous swelling materials or complex deformations. Deformation measurement has also turned out to be challenging near the edges of the sample. In these situations, DECT has the potential to be more accurate, which is supported by the results shown in Fig. 5, especially near the water inlet. Additionally, the reference CT method relies on a known water content distribution in a chosen reference state (usually the initial state), which also serves as the reference state for deformation measurement. DECT does not require this prior knowledge about the distribution of water content, increasing its applicability.

Future research should focus on reducing noise levels, improving the accuracy of MD, and increasing the information obtained by using DECT (or spectral CT). This involves optimising imaging protocols and employing advanced noise reduction filters. This study has focused on empirical corrections of raw data, and the MD was performed with a relatively simple voxel-wise approach. However, suitable prior information can be incorporated into the MD using regularisation techniques. A promising direction is performing energy discrimination using PCDs. These detectors facilitate simultaneous image acquisition across energy bins, enabling flexible adjustments to those bins and potentially enhancing the ability to separate, identify, and quantify materials. Simultaneously acquiring images across different energy bins prevents displacement issues in DECT MD, making the development of PCDs crucial for effective DECT-based process monitoring. Furthermore, using PCDs would also help in selecting independent energy intervals for the two spectra required in DECT with greater accuracy than selecting spectra with tube voltage and filtering. The low energy levels would be preferable for MD, as variations in mass attenuation coefficients are primarily due to the photoelectric effect (see Fig. 2b), and the impact of the photoelectric effect on total attenuation decreases with increasing energy. However, sufficient transmittance through the sample gives a lower limit for the usable energies, and therefore, smaller samples could enable higher accuracy measurements in DECT MD.

This study focused on two main components without a detailed analysis of the mineralogical composition. The classification of voxels was useful for removing unrealistic density values, but it should be further developed to identify minerals. DECT, using calibration with known composition samples or using techniques that provide chemical information, can offer insights into the mineralogy of multi-material geological samples^55,56^. One interesting method for chemical identification of minerals, particularly well-suited for PCDs, involves detecting element-specific discontinuities known as K-edges in the LAC^57^. However, K-edges are not always present in the imaging energy range. A generally applicable approach identifies the effective atomic number and mass, or electron, density of the sample with the unknown composition. Despite technological progress, quantitative material characterisation requires consideration of system- and sample-specific factors, as well as image artefacts^55–57^.

## Conclusions

DECT post-reconstruction MD proved to be a viable tool for quantitative two-phase density analysis in bentonite during water infiltration despite higher noise levels and discrepancies in water content values compared to reference methods. A two-step beam hardening correction and scatter correction using a beam-stop array were vital parts of the analysis. Future research should focus on reducing noise levels and enhancing DECT accuracy through optimised imaging protocols and more advanced image processing and analysis methods. Performing energy discrimination on the detector side could unlock new possibilities. Overall, DECT, or spectral CT in general, is suitable for investigating the spatio-temporal distributions of components in bentonite-water mixtures.

## Data Availability

The datasets generated during and/or analysed during the current study are available from the corresponding author on reasonable request.
